# MS-TSEFNet: Multi-Scale Spatiotemporal Efficient Feature Fusion Network

**DOI:** 10.3390/s26020437

**Published:** 2026-01-09

**Authors:** Weijie Wu, Lifei Liu, Weijie Chen, Yixin Chen, Xingyu Wang, Andrzej Cichocki, Yunhe Lu, Jing Jin

**Affiliations:** 1 Key Laboratory of Smart Manufacturing in Energy Chemical Process, Ministry of Education, East China University of Science and Technology, Shanghai 200237, China; weijiewu203096@foxmail.com (W.W.); toravo_llf@outlook.com (L.L.); wjchen827@foxmail.com (W.C.); yixinchen1022@foxmail.com (Y.C.); xywang@ecust.edu.cn (X.W.); 2 Systems Research Institute of Polish Academy of Sciences, 01-447 Warsaw, Poland; cichockiand@gmail.com; 3 Faculty of Physics, Astronomy and Applied Computer Science, Nicolaus Copernicus University (UMK), 87-100 Torun, Poland; 4 RIKEN Advanced Intelligence Project, Tokyo 103-0027, Japan; 5 Institute of Global Innovation Research, Tokyo University of Agriculture and Technology, Tokyo 184-8588, Japan; 6 Shanghai Lansheng Brain Hospital Investment Co., Ltd., Shanghai 200336, China; 7 School of Mathematics, East China University of Science and Technology, Shanghai 200237, China

**Keywords:** motor imagery, brain–computer interface, convolutional neural networks

## Abstract

Motor imagery signal decoding is an important research direction in the field of brain–computer interfaces, which aim to judge the motor imagery state of an individual by analyzing electroencephalogram (EEG) signals. Deep learning technology has been gradually applied to EEG classification, which can automatically extract features. However, when processing complex EEG signals, the existing decoding models cannot effectively fuse features at different levels, resulting in limited classification performance. This study proposes a multi-scale spatiotemporal efficient feature fusion network (MS-TSEFNet), which learns the dynamic changes in EEG signals at different time scales through multi-scale convolution modules and combines the spatial attention mechanism to efficiently capture the spatial correlation between electrodes in EEG signals. In addition, the network adopts an efficient feature fusion strategy to deeply fuse features at different levels, thereby improving the expression ability of the model. In the task of motor imagery signal decoding, MS-TSEFNet shows higher accuracy and robustness. We use the public BCIC-IV2a, BCIC-IV2b and ECUST datasets for evaluation. The experimental results show that the average classification accuracy of MS-TSEFNet reaches 80.31%, 86.69% and 71.14%, respectively, which is better than the current state-of-the-art algorithms. We conducted an ablation experiment to further verify the effectiveness of the model. The experimental results showed that each module played an important role in improving the final performance. In particular, the combination of the multi-scale convolution module and the feature fusion module significantly improved the model’s ability to extract the spatiotemporal features of EEG signals.

## 1. Introduction

As a communication and control technology with great potential, brain–computer interfaces (BCIs) have made significant progress in recent years [1,2,3,4,5]. BCI technology realizes control intention prediction, psychological state detection and other functions by analyzing brain neural activity signals and extracting information, providing new possibilities for medical rehabilitation, intelligent interaction and neuroscience research. In the field of non-invasive technology, electroencephalogram (EEG) signals are widely used to record neural electrical activity due to their advantages such as high temporal resolutions, portability and low costs. EEG-based BCI paradigms include the steady-state visual evoked potential (SSVEP) [6,7,8], the event-related potential (ERP) [9,10], etc. Among them, the motor imagery (MI) paradigm [11] has become one of the research hotspots by guiding users to imagine specific tasks and decode their intentions. Motor imagery has been successfully applied to fields such as smart homes [12], rehabilitation medicine [13] and entertainment [14]. It not only provides new control methods for people with disabilities but also promotes innovations in virtual reality and game interaction.

However, EEG signals have the characteristics of low spatial resolution, susceptibility to noise interference, and large individual differences, resulting in lower accuracy of motor imagery decoding than other BCI technologies. In early studies, researchers mainly used classical machine learning methods to classify EEG signals. Common spatial pattern (CSP) [15] uses linear transformation to find a set of spatial filters to maximize the variance between different motor imagery tasks to enhance the discrimination between tasks. Fast Fourier transform (FFT) [16] converts EEG signals from time domain to frequency domain to extract frequency information in the signal. Wavelet transform (WT) [17] extracts local information of the signal at different scales by continuously convolving the signal with wavelet functions of different frequencies. Based on the above feature extraction methods, researchers use classifiers such as support vector machine (SVM) [18], multilayer perceptron (MLP) [19,20], and linear discriminant analysis (LDA) [21] to decode EEG signals. However, these traditional methods rely on manual feature extraction and are difficult to fully explore the complex spatiotemporal features in EEG signals, limiting the further improvement of classification performance.

In recent years, with the rise in deep learning, methods such as convolutional neural networks (CNN) [22] and long short-term memory networks (LSTM) [23] have gradually become important tools for processing EEG signals, especially in large-scale data and complex tasks, showing stronger adaptability and accuracy. R. T. Schirrmeister et al. [24] used CNN to directly learn effective features from raw EEG data, overcoming the tedious process of manually extracting features in traditional methods. EEGNet [25] can efficiently capture the time domain and frequency domain information in EEG signals by designing an optimized convolutional layer structure, avoiding complex preprocessing steps, and performing well in various EEG signal classification tasks. EEGTCNet [26] introduced a temporal convolutional network (TCN), which is designed specifically for processing sequence data with long-term dependencies. TCN can capture dependencies over a longer time range by expanding the receptive field of the convolution kernel and is not easily affected by gradient vanishing, thereby effectively improving the performance and stability of EEG signal processing. By integrating information from both the time and frequency domains via an innovative frequency interaction mechanism, IFNet [27] is capable of more effectively extracting motor imagery-related features from EEG signals. EEG Conformer [28] extracts rich time domain and frequency domain information from EEG signals by fusing CNN and Transformer, and deeply models long time series, further improving classification performance.

Although deep learning technology has made significant progress in EEG decoding, it still faces many challenges. First, EEG signals are easily interfered by noise and artifacts, resulting in low signal-to-noise ratio and affecting decoding accuracy. Second, EEG signals have strong time dependence and dynamic changes, and existing decoding methods are difficult to effectively capture this time series information. In addition, feature selection and high dimensionality problems increase the computational complexity of the decoding model, and overfitting is prone to occur during training. The diversity and real-time requirements of decoding tasks also further increase the complexity of the system. Especially with multi-channel and large-scale data, computational efficiency and latency become bottlenecks restricting real-time applications.

Against the above challenges, this study focuses on four core problems that the proposed MS-TSEFNet specifically addresses: (1) Existing models lack effective multi-scale temporal feature extraction mechanisms, failing to comprehensively capture the dynamic changes in EEG signals across different time scales. (2) The fusion of multi-level features (shallow vs. deep, spatial vs. temporal) is overly simplistic, unable to adaptively enhance task-relevant features and suppress noise. (3) Most state-of-the-art methods sacrifice either classification accuracy for computational efficiency or vice versa, lacking a balanced solution that meets both practical accuracy and real-time application requirements. (4) The ability to suppress noise and artifacts in EEG signals is insufficient, leading to unstable feature representation.

To address these specific problems, this paper proposes a multi-scale spatiotemporal efficient feature fusion network (MS-TSEFNet) based on multi-attention: specifically, its multi-scale temporal convolution module with parallel kernels captures transient, rhythm-related, and long-term temporal features to solve the problem of incomplete multi-scale feature extraction, while the EFF module integrating EAG, ECA, and SA realizes adaptive fusion of multi-level features to address the limitation of simplistic feature fusion. Additionally, through structural optimization and efficient attention mechanisms, the model achieves a favorable balance between accuracy and computational cost, as verified by its training time comparable to state-of-the-art methods. Furthermore, the SE module and spatial attention mechanism work jointly to suppress noise and enhance task-relevant features, effectively improving the robustness of feature representation. The main contributions of this paper are as follows:

1. A deep learning architecture is proposed to enhance the spatiotemporal representation ability of motor imagery tasks by exploring the interaction of features at different levels and refine the feature selection at different scales. The architecture combines multi-scale convolution modules and features fusion modules, which can extract rich spatiotemporal features from EEG signals and improve the expression ability of the model through efficient fusion strategies.
2. The enhanced attention gating mechanism is introduced, including channel attention and spatial attention, which effectively improves the signal decoding ability and the robustness of the model. The channel attention mechanism enhances the model’s attention to important features by adaptively adjusting the weights of feature channels; the spatial attention mechanism further enhances the model’s ability to extract spatial features of EEG signals by capturing the spatial correlation between electrodes.
3. Comprehensive experimental verification was carried out on two public benchmark datasets (BCIC-IV2a and BCIC-IV2b) and one private dataset (ECUST Dataset). The results showed that the average classification accuracy of MS-TSEFNet reached 80.31%, 86.69% and 71.14%, respectively, which is better than the most advanced algorithms. In addition, the contribution of each module to the classification performance was further demonstrated through ablation experiments, verifying the effectiveness and superiority of the proposed method. The ablation experimental results show that the combination of multi-scale convolution module and feature fusion module significantly improves the model’s ability to extract spatiotemporal features of EEG signals, while the introduction of attention mechanism further enhances the model’s robustness and generalization ability.

## 2. Materials and Methods

### 2.1. Dataset Description

In this study, two public benchmark datasets and one private dataset were used to evaluate the performance of the baseline model and MS-TSEFNet. These datasets were collected under different experimental settings, with varying numbers of subjects and sample sizes, covering a variety of motor imagery tasks and EEG signal acquisition conditions. The details are as follows:

1. BCI Competition IV 2a Dataset (BCIC-IV2a): This dataset consists of 22 Ag/AgCl electrodes with a sampling rate of 250 Hz and records the motor imagery task data of 9 healthy subjects. Subjects perform four tasks, including motor imagination of the left hand, right hand, foot, and tongue. Each subject had two sessions on different days, each session containing 6 runs, and each run containing 48 trials. At the beginning of each trial, the subject will see a fixed cross, followed by a cue arrow indicating a motor task that requires imagination. Subjects begin imagining within 1.25 s of cue appearance for 4 s. The dataset contains a total of 9 subjects × 2 sessions × 6 runs × 48 trials = 5184 trials, providing rich data support for the quadruple classification task.
2. BCI Competition IV 2b Dataset (BCIC-IV2b): This dataset consists of 9 subjects performing 2 types of motor imagery (left-handed vs. right-handed) at a sampling rate of 250 Hz. The three electrodes are located at positions C3, Cz, and C4 in the International 1020 system, which are critical areas for the motor imagery task. Each subject conducts 5 sessions with no feedback recorded for the first two sessions and feedback for the last three times. Each session contains 10 runs, and each run contains 20 trials. The experimental procedure is similar to that of BCI-IV2a, but the duration is shorter, prompting the initiation of imagination 3 s after presentation and lasting 3.5 s. The dataset contains a total of 9 subjects × 5 sessions × 10 runs × 20 trials = 9000 trials, providing a large sample for dichotomous tasks.
3. ECUST Dataset: EEG data were collected from 11 healthy subjects (8 males and 3 females), ranging in age from 23 to 27 years. All subjects met the following criteria: normal visual acuity or corrected to normal visual acuity, no major medical illness, no history of psychiatric illness. Before data collection, the experimenter will explain the experimental process to the subjects in detail and ask them to sign an informed consent form. In order to ensure the quality of the data, the subjects were required to keep their bodies relaxed during the experiment and avoid blinking, swallowing and other actions that may produce electromyography artifacts. This research protocol (Ethics Approval No.: ECUST-2022-054) strictly follows the relevant provisions of the Declaration of Helsinki, and all experimental procedures have been reviewed by the Biomedical Ethics Committee of East China University of Science and Technology.

### 2.2. EEG Data Preprocessing

In the pre-processing phase, we systematically processed the EEG signals to ensure data quality and consistency. For channel selection, we used an optimization strategy for different datasets: in the BCIC-IV2a dataset, we used all 22 Ag/AgCl electrode channels, which cover a wide area of the brain and are able to fully capture the neural activity associated with the motor imagery task. For the BCIC-IV2b dataset, we used 3 key channels located at the C3, Cz, and C4 positions, which correspond to areas of the brain’s motor cortex and are the most informative areas of the motor imagery task.

In order to ensure the consistency of the sampling rate between different datasets, we used a sampling rate of 250 Hz for all data. In terms of temporal selection, we extracted data segments from 0.5 to 3.5 s after cue onset in each trial, which contains key neural responses to the motor imagery task, while avoiding the visual evoked potentials at the time of cue occurrence and extraneous neural activities after the end of the task.

For frequency domain processing, we used a 0.5–40 Hz bandpass filter, a choice based on both the physiological characteristics of MI-EEG signals and the architectural design of MS-TSEFNet. This frequency range comprehensively covers the core rhythmic activities associated with MI tasks—specifically the mu rhythm (8–12 Hz) and beta rhythm (13–30 Hz), which are well-documented to reflect neural activity related to motor planning and execution. Additionally, the low cutoff (0.5 Hz) removes slow drift artifacts, while the high cutoff (40 Hz) suppresses high-frequency noise (e.g., electromyographic interference), ensuring the retention of task-relevant features without redundant noise. Notably, we did not adopt adaptive band selection or Filter Bank approaches (e.g., FBCNet), as this decision aligns with MS-TSEFNet’s core design philosophy: leveraging multi-scale temporal convolution to inherently capture frequency-specific features, rather than relying on preprocessing-level frequency decomposition.

### 2.3. Model Architecture

**Figure 1:**
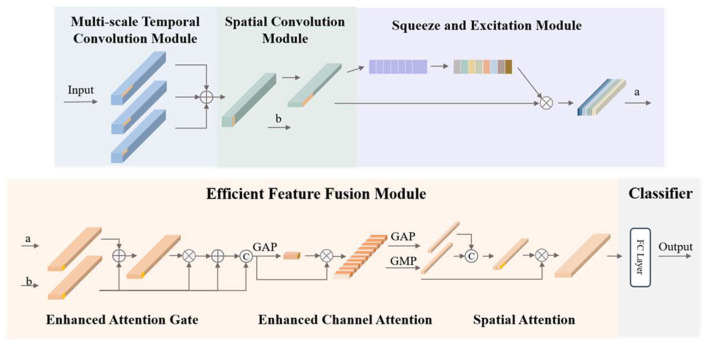
The overall structure of MS-TSEFNet, including multi-scale temporal convolution module, spatial convolution module, squeeze and excitation module, efficient feature fusion module and classifier. Symbol a and b represent the upper module output and subsequent module input.

In this section, we propose a novel multi-scale spatiotemporal efficient feature fusion network (MS-TSEFNet), which consists of a multi-scale temporal convolution module, a spatial convolution module, a Squeeze-and-Excitation (SE) module, an Efficient Feature Fusion (EFF) module, and a classifier, as shown in Figure 1. The network adopts a cascade structure, where preprocessed EEG data is fed into the multi-scale temporal convolution module sequentially, spatial convolution module, SE module, EFF module, and finally enters the classifier for classification. The architecture of MS-TSEFNet was systematically selected and optimized through a series of experimental validations to ensure its adaptability to MI-EEG decoding. The integration of spatial convolution, SE module, and EFF module was validated through ablative experiments (Section 3.2). We tested multiple module combinations and confirmed that the synergistic effect of these modules significantly improves feature representation and noise suppression. Finally, the cascade order of modules was optimized by comparing different sequences, ensuring that multi-scale temporal features are first extracted, then spatial correlations are modeled, and finally feature enhancement. This structural design fully considers the spatiotemporal characteristics of EEG signals, and can effectively extract multi-level feature representations.

#### 2.3.1. Multi-Scale Temporal Convolution Module

The multi-scale temporal convolution module is one of the core components of MS-TSEFNet, which aims to extract features of EEG signals from different time scales. The module uses a parallel convolution structure, using three convolutional kernels of different sizes (13, 33, 67) to process the input signal simultaneously. We initially tested a range of candidate sizes (11, 13, 25, 33, 51, 67, 128) on the BCIC-IV2a dataset and confirmed this trio optimally balances feature coverage and computational efficiency. Smaller convolutional kernels (size 13, ~52 ms at 250 Hz sampling rate) capture rapidly changing transient features, matching the upper bound of the beta rhythm (13–30 Hz) period; the medium kernel (size 33, ~132 ms) is tailored for extracting mu (8–12 Hz) and beta rhythm-related motor imagery features, covering the full period of mu rhythm and multiple cycles of beta rhythm; larger kernels (size 67, ~268 ms) capture slower rhythm changes and long-term dependencies of mu/beta dynamics. Through multi-scale feature extraction, the model comprehensively characterizes the temporal dynamics of EEG signals. Each convolutional layer is followed by a batch normalization and *ReLU* activation function to improve the training stability and nonlinear expression ability of the network.

#### 2.3.2. Spatial Convolution Module

The spatial convolution module processes the electrode dimension through convolution operations, and effectively captures the spatial correlation between different electrodes. The module adaptively extracts task-relevant spatial patterns by linearly combining multi-channel EEG signals through a learnable weight matrix. Specifically, for input multichannel EEG features, the spatial convolutional layer performs a feature transformation of the electrode dimension, where each output channel is a weighted combination of all input channels. This design allows the model to automatically learn the functional connection patterns between the electrodes, which is particularly useful for identifying the contralateral activation patterns typical of motor imagery tasks.

To prevent the model from learning non-physiological spatial correlations induced by the fixed electrode layout (e.g., spurious correlations arising from physical electrode adjacency rather than underlying neural activity coordination), two core constraint mechanisms are integrated into the module pipeline. First, the convolution kernel of the global spatial convolution layer is initialized with weak, near-zero weights and optimized alongside batch normalization; this setup normalizes the feature distribution across electrodes, effectively suppressing overfitting to layout-specific noise that does not reflect neural physiology. Second, the subsequent SE module dynamically recalibrates the channel weights corresponding to different electrodes—strengthening the weights of electrodes with established physiological relevance to motor imagery and weakening those of electrodes that primarily carry non-physiological layout-driven correlations. This dual constraint ensures that the global spatial convolution module focuses on learning neurophysiologically meaningful spatial patterns rather than non-physiological artifacts derived from the fixed electrode arrangement.

#### 2.3.3. Squeeze-and-Excitation Module

The SE module dynamically adjusts the channel weights of the feature map through the channel attention mechanism. Firstly, the spatial information of each channel is compressed into a single scalar through global average pooling (Squeeze operation), and then the nonlinear relationship between channels is learned through two fully connected layers (Excitation operation) to generate channel weight vectors, where the reduction ratio of the SE module is set to 16. This reduction ratio means the number of neurons in the first fully connected layer is reduced to 1/16 of the input channel number, which balances computational efficiency and feature representation ability. These weights are used to recalibrate the channel importance of the feature map, enhance the response to task-related features, and suppress noisy or irrelevant features. In the EEG decoding task, the SE module can automatically identify the frequency bands and spatial patterns that are most relevant to the motor imagery, improving the quality of feature representation.

#### 2.3.4. Efficient Feature Fusion Module

The EFF module combines three important mechanisms, Enhanced Attention Gate (EAG), Efficient Channel Attention (ECA), and Spatial Attention (SA), to improve the representation ability and decoding accuracy of complex EEG signals in EEG decoding systems. The Enhanced Attention Gate module is a feature fusion method based on attention mechanism, which aims to dynamically adjust the weights of two input features a and b to enhance task-related features and suppress noise or irrelevant information. The core idea of the EAG module is to generate weights through a lightweight attention mechanism and apply them to the input feature b, so as to achieve adaptive enhancement of features. Firstly, the feature transformation of deep input a and shallow input b is carried out to extract the key information of the input features, which provides the basis for the subsequent attention weight generation, and the calculation process of $W_{a}$ and $W_{b}$ is as follows:

(1) $$W_{a}\;=\;ReLU\left(BN\left(GroupConv\left(a\right)\right)\right),$$

(2) $$W_{b}=ReLU\left(BN\left(GroupConv\left(b\right)\right)\right),$$

Among them, *ReLU* is the activation function, *BN* is batch normalization, and GroupConv is the group convolution with the number of groups set to 32. The grouped convolution layers adopt a kernel size of 1 × 1, stride of 1, padding of 0, and bias = True. The mathematical expression for the EAG module is as follows:

(3) $$EAG\left(a,\;b\right)\;=\;b\times \left(1+Sigmoid\left(Conv\left(ReLU\left(W_{a}+W_{b}\right)\right)\right)\right),$$

where Sigmoid and ReLU are activation functions, and Conv is a 1 × 1 convolution operation for further extracting attention weights.

The Efficient Channel Attention module is designed to optimize channel-level feature representation through a lightweight attention mechanism. The core idea of the ECA module is to capture the global information between channels through global pooling operations, and use convolution operations to generate channel attention weights, so as to enhance the response of task-dependent channels while suppressing noise or unrelated channels. ECA performs residual concatenation of the GAP feature map, and then performs global mean pooling (GAP) and global maximum pooling (GMP) to capture the global and local salient information between channels. Global average pooling generates a channel-level descriptor by averaging the spatial dimensions of the feature map, reflecting the global response strength of each channel. Global maximum pooling captures salient features within the channel by extracting the maximum value of each channel. Next, the ECA module stitches together the results of GAP and GMP to form a comprehensive channel descriptor. This descriptor contains global and local salient information between channels, which provides rich input for subsequent attention weight generation. The ECA module transforms the spliced channel descriptor through convolution operation to generate channel attention weights. Finally, the ECA module multiplies the generated channel attention weights with the input feature maps one by one to realize the reweighting of the feature maps. In this way, the ECA module is able to dynamically enhance the response of task-relevant channels while suppressing interference from noise or unrelated channels.

The Spatial Attention (SA) module is used to capture the spatial dependencies of feature maps and enhance the spatial regions related to the task. The core parameters are set as follows: the convolution kernel size is 7, with padding = 3; the convolutional layer (conv1) takes 2 input channels (concatenation of average-pooled and max-pooled features) and outputs 1 channel. The feature map after channel attention optimization is spliced with the original input feature map to form a comprehensive spatial feature map. Specifically, the module first computes the average-pooled feature map and max-pooled feature map along the channel dimension, then concatenates them to form a 2-channel feature map. This stitching operation preserves the details of the original feature map and introduces the optimized channel features of the ECA module. Next, the SA module transforms the spliced spatial feature map through a 2D convolution operation to generate spatial attention weights. The resulting attention weights are normalized by the Sigmoid function. Finally, the SA module multiplies the generated spatial attention weights with the output of the ECA module element by element to realize the spatial weight weighting of the feature map. In this way, the SA module is able to dynamically enhance the response of important spatial regions while suppressing interference from irrelevant areas.

Although the spatial information and channel information of the EEG signal are related to some extent, they have a different focus. The spatial information reflects the spatial distribution and functional connections between the electrodes, while the channel information reflects the time-frequency characteristics recorded by each electrode. In MS-TSEFNet, the ECA module and the SA module are used to process channel information and spatial information, respectively, and the spatiotemporal feature extraction ability of the model for EEG signals is significantly improved through the hierarchical feature extraction and attention mechanism. This design allows MS-TSEFNet to adaptively focus on the most informative part of the EEG signal, thus providing strong support for tasks such as motion imaging classification.

The classifier uses the fully connected layer and the softmax function to map the extracted spatiotemporal features to the corresponding motor imagery categories. Through end-to-end training, MS-TSEFNet can automatically learn the mapping relationship from the original EEG signal to the motion imagination category, which greatly reduces the workload of manual feature engineering while ensuring the performance of the model.

### 2.4. Training Process

We use 5-fold cross validation and adopt the two-stage training strategy mentioned in [27,29]. For the 5-fold cross validation, the dataset of each subject is randomly divided into 5 mutually exclusive subsets with equal sample size. During the training process, 4 subsets are used as the training set to train the model, and the remaining 1 subset serves as the validation set to evaluate the model’s generalization performance. This process is repeated 5 times, with each subset used as the validation set exactly once, ensuring that every sample participates in both training and validation. MI EEG signals exhibit strong inter-subject heterogeneity and limited sample size per subject, making traditional single train-test splitting prone to overfitting or biased performance estimation due to accidental data distribution. As a standard validation protocol in BCI research [24,25], 5-fold cross validation maximizes the utilization of limited data, reduces random fluctuations caused by partition bias, and provides a more robust evaluation of the model’s generalization ability, which is crucial for verifying the stability of MS-TSEFNet in decoding EEG signals with individual differences.

For the two-stage training strategy: In the first stage, we train the network on the training set and save the model with the minimum validation loss. In the second stage, we fine-tune the network using all the data. When the training loss of the second stage is lower than the training loss of the first stage, the training process stops.

We implemented the model using the PyTorch 2.5.1 library on Python 3.10 and the GPU is 4060. All core modules were implemented from scratch to match the proposed architectural design, without relying on ready-made software or pre-built network templates. For the baseline models, we adapted and reproduced their open-source implementations to ensure consistent experimental settings across all comparisons. These baseline implementations were integrated into our custom code framework, with necessary adjustments to align with the input format and training pipeline of MS-TSEFNet, avoiding biases caused by using disjoint software tools.

During the training process, cross entropy loss and AdamW [30] optimizer are used to continuously update the network parameters. During the training process, the key hyperparameters are set as follows: batch size is 32; initial learning rate is 2 × 10^−12^, and a cosine annealing learning rate scheduler is adopted to adjust the learning rate dynamically (the minimum learning rate is 1 × 10^−6^); weight decay of AdamW optimizer is 0.01. The first stage is trained for 1000 epochs, and the second stage is trained with an early stopping strategy. The overall effect of the model is measured by accuracy and Kappa value. Accuracy quantifies the proportion of correctly classified trials relative to the total number of trials and provides an intuitive reflection of overall classification performance. The Kappa coefficient measures the agreement between the model’s predictions and the true labels while eliminating the impact of random guessing. Relying solely on accuracy cannot fully reflect the model’s stability, and the Kappa coefficient complements accuracy by quantifying the model’s discriminative power beyond chance, making the evaluation more comprehensive and objective. The calculation formulas for accuracy and Kappa are as follows:

(4) $$Acc\;=\;\frac{TP+TN}{TP+FP+TN+FN}$$

(5) $$Kappa=\frac{p_{o}-p_{e}}{1-p_{e}}$$

where TP is a true positive, TN is a true negative, FP is a false positive, FN is a false negative, po is the average accuracy over all trials and pe is the accuracy of random guessing.

### 2.5. Baseline Model

1. EEGNet: EEGNet proposed by Lawhern et al. [25] uses deep separable convolution and optimization techniques to reduce computational complexity while maintaining high classification performance, making it particularly suitable for tasks such as motor imagery and emotion recognition. Due to its high efficiency and small number of parameters, EEGNet has become a widely used benchmark model that helps evaluate the performance of new algorithms.
2. FBCNet: FBCNet proposed by R. Mane et al. [29] is an improved model based on EEGNet. It introduces filter bank technology, which enables the model to extract features in multiple frequency bands simultaneously, thereby enhancing the learning ability of different frequency bands in EEG signals. Through cross-band feature fusion, FBCNet can better capture the time-frequency information in EEG signals and improve classification accuracy.
3. EEG Conformer: The EEG Conformer [28] combines a convolutional neural network (CNN) and a self-attention mechanism. By introducing a self-attention mechanism similar to the Transformer, the long-distance dependencies and spatiotemporal characteristics in EEG signals are fully considered. On the basis of traditional CNNs, the relationship between time steps is captured through the self-attention layer, thereby improving the model’s ability to model complex EEG patterns.
4. ADFCNN [31] is a dual-scale fusion convolutional neural network based on the attention mechanism. The model extracts local and global features of EEG signals at different scales and weightedly fuses these features through the attention mechanism to achieve dual-scale fusion.

## 3. Results

### 3.1. Comparison with Baseline Models

In this study, we compared the classification accuracy performance of four different methods (EEGNet, FBCNet, Conformer, ADFCNN) on two publicly available datasets (BCIC-IV2a and BCIC-IV2b) and one private dataset (ECUST Dataset). Table 1 shows the comparison of MS-TSEFNet with other methods on the BCIC-IV2a dataset. It can be seen that our method outperformed all other methods in terms of average accuracy (80.31%), especially in the results of subjects 1, 2, 6, 8, and 9. The standard deviation (11.66%) was lower than that of other methods (e.g., FBCNet and EEGNet), indicating that the stability of our model between different subjects was also improved.

Table 2 shows the comparison of MS-TSEFNet with other methods on the BCIC-IV2b dataset. MS-TSEFNet also showed strong competitiveness on this dataset, especially in subject 3. In terms of average accuracy (86.69%), MS-TSEFNet ranked first among all methods and had the lowest standard deviation (8.24%), indicating that the model had high stability in its performance among different individuals.

**Figure 2:**
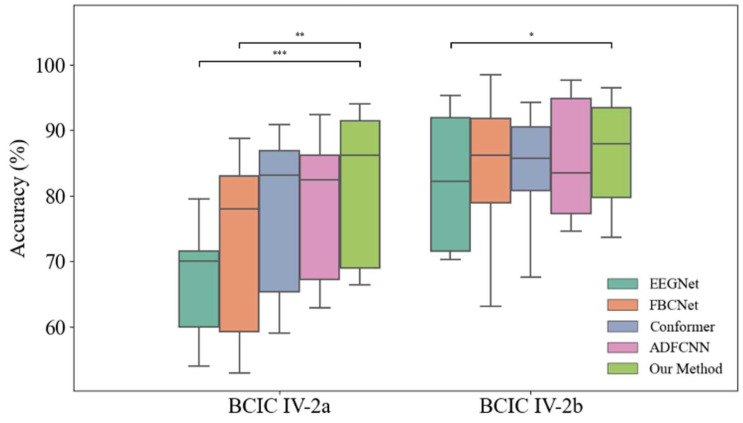
Comparison of the classification performance of the proposed method with other methods on BCIC-IV2a and BCIC-IV2b datasets.

In order to further verify the superiority of our method, we performed a statistical analysis of the significant differences between the different methods, specifically a two-tailed independent samples *t*-test. This test was conducted based on the classification accuracy of all subjects across different models, with the prerequisites of normality (verified via Shapiro–Wilk test) and homogeneity of variance (confirmed through Levene’s test) satisfied, as shown in Figure 2. In the BCIC-IV2a dataset, there was a significant difference between Our method and EEGNet (*p* < 0.001, marked with ***), and significant differences from FBCNet (*p* < 0.01, marked with **). For EEG Conformer and ADFCNN, although there was no significant difference, the mean classification was better. In the BCIC-IV2b dataset, the difference between Our Method and EEGNet was equally significant (*p* < 0.05, marked with *).

As shown in Table 3, our method achieves the highest average classification accuracy of 71.14%, outperforming all competing methods. Notably, our model exhibits superior robustness, as evidenced by its competitive standard deviation (13.02), second only to ADFCNN (11.52), suggesting more stable performance among subjects. Further analysis showed that our method consistently performed well in challenging subjects (e.g., Sub1: 62.80%, Sub4: 83.33%, Sub7: 67.50%, Sub8: 73.50%) while mitigating the severe performance degradation observed in other models (e.g., EEGNet was 56.83% in Sub7). While the EEGNet and ADFCNN baselines are strong, the improvements to EEGNet and ADFCNN highlight the effectiveness of our architectural innovations in capturing discriminative EEG features.

### 3.2. Ablation Study

In order to verify the contribution of each module in the model to the final performance, we conducted an ablation experiment using the BCIC IV2a dataset. In the experiment, we replaced the multi-branch structure with the single-branch result, deleted the SE module and deleted the EFF module, and explored the effects of these operations on the model individually and simultaneously. The results are shown in Table 4.

The experimental results show that all three operations will lead to a decrease in the classification performance of the model. In the experiment of a single operation, replacing the multi-branch structure caused the average classification accuracy to drop by 4.35%. This may be because the multi-branch structure originally extracted different temporal features of the signal through multiple convolution kernels in parallel, while the single-branch structure only used a single convolution kernel, which could not fully capture the temporal dependency and complex features of the signal, thus affecting the performance of the model. After deleting the SE module, the classification accuracy dropped to 76.99%. This decline may be because the SE module weighted the features of each electrode channel, allowing the model to focus on more important signal channels. When the SE module was deleted, the weights of all electrode channels became the same, causing the model to ignore the signal differences between different electrode channels, thereby reducing the classification effect. After deleting the EFF module, the classification accuracy further dropped to 75.77%, which had the greatest impact on the model classification performance in the single-branch experiment. This may be because the EFF module effectively integrates multiple feature spaces, thereby enhancing the model’s feature representation ability. When the EFF module is deleted, the model cannot fully integrate information from different feature spaces, resulting in a weakened ability to express complex data, affecting the final classification effect.

In the experiment of replacing or removing multiple modules at the same time, when the MTC multi-branch structure is replaced and the EFF module is removed at the same time, the average classification accuracy of the model decreases by 3.61%. When the MTC multi-branch structure is replaced and the SE module is removed, the average classification accuracy of the model decreases by 4.19%. When the EFF module and the SE module are replaced at the same time, the average classification accuracy decreases by 3.25%. When all three modules are operated, the average classification accuracy of the model is 74.69%, a decrease of 5.71%. From the above experiments, it can be seen that replacing or removing certain components will lead to a significant decrease in performance, and the performance of the model fluctuates greatly. We further prove the irreplaceability of each module in the task.

**Figure 3:**
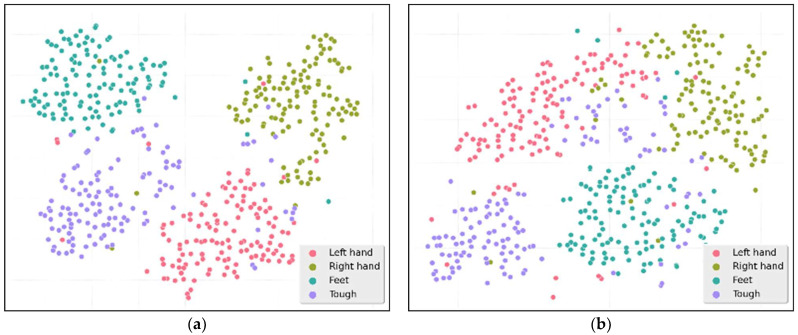
t-SNE visualization of fusion features: (**a**) four-class stitched distribution of fusion features of Subject 1 in the BCIC-IV2a dataset, (**b**) four-class attention distribution of fusion features of Subject 1 in the BCIC-IV2a dataset after the MTC&SE&EFF module.

The t-SNE visualization in Figure 3 illustrates the critical role of the MTC&SE&EFF module in maintaining the discriminative feature distribution. While the full model (a) shows well-separated clusters of all four classes, removing module (b) results in a significant increase in overlap between classes, especially between left-handed and right-handed movements. The Feet and Tongue classes also exhibit a more dispersed distribution and smaller interclass margins. This increase in confusion in the feature space directly corresponds to the performance degradation we observed in our ablation studies, confirming that the integration module is critical to maintaining the uniqueness of the motion-related features in the BCIC-IV2a dataset.

However, it is necessary to note a key limitation of t-SNE: its results are highly sensitive to hyperparameter choices (e.g., perplexity, number of iterations). In our analysis, we set the perplexity to 30 (a widely adopted value for EEG feature visualization) and the number of iterations to 1000—these settings are consistent with common practices in BCI-related feature visualization, but variations (e.g., lower perplexity leading to overly compact clusters, insufficient iterations causing unstable sample distributions) could alter the observed clustering patterns. Thus, the t-SNE results here should be interpreted as a qualitative auxiliary reference rather than a quantitative measure of feature discriminability.

### 3.3. Confusion Matrix

**Figure 4:**
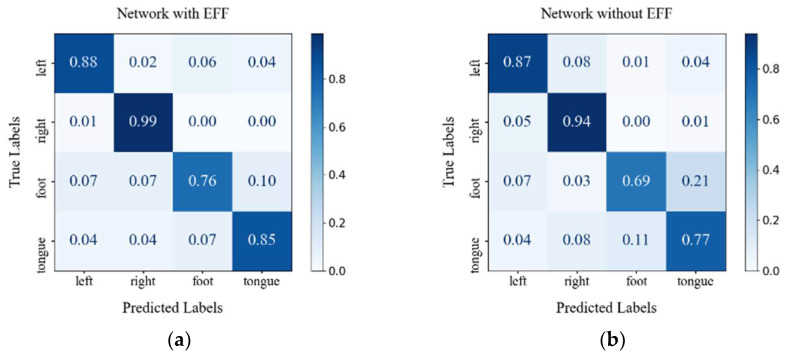

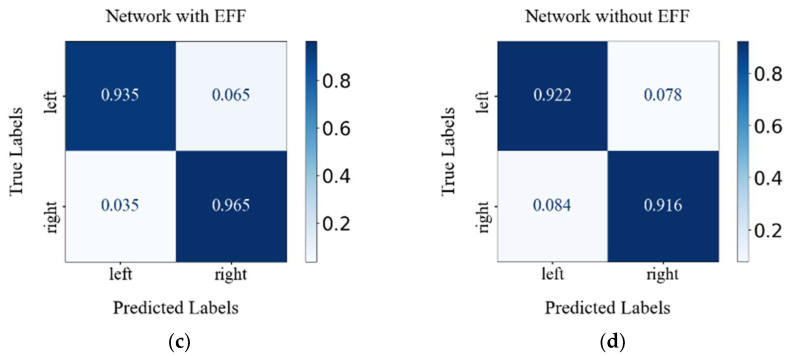
Confusion matrices for proposed networks with and without Efficient Feature Fusion Module (EFF) (**a**) BCIC-IV2a dataset (Subject 1) with EFF; (**b**) BCIC-IV2a dataset (Subject 1) without EFF; (**c**) BCIC-IV2b dataset (Subject 5) with EFF; (**d**) BCIC-IV2b dataset (Subject 5) without EFF.

To further illustrate the effectiveness of the EFF module for classification, Figure 4 presents the confusion matrices of the model on the BCIC-IV2a and BCIC-IV2b datasets before and after the integration of the EFF module. As can be seen from Figure 4; after adding the EFF module, the correct classification rate of the left hand increased from 87% to 88%, and that of the right hand increased from 94% to 99%. Although the improvement is modest, it indicates that the EFF module has a positive effect on enhancing the classification ability for left-hand and right-hand motor imagery. For the foot and tongue motor imagery classification tasks, the correct classification rate increased by more than 7% after integrating the EFF module. This may be because the EFF module better captures and fuses feature information related to foot and tongue motor imagery, further reducing the misclassification rate.

For the two-classification task, after adding the EFF module, the accuracy of the model increased from 91.9% to 95%, which shows that the addition of the EFF module improves the overall classification performance of the model. In the left-hand classification task, the correct rate increased by 1.3%, and the EFF module has a certain effect on improving the classification performance of the left hand. In the right-hand classification task, the correct classification rate increased to 96.5%, an increase of 4.9%. This change indicates that the EFF module has a more significant lifting effect on the right hand.

### 3.4. The Effect of Time Window

To explore the impact of different time windows on model classification accuracy and the information transfer rate (ITR), we conducted a sensitivity analysis on the BCIC-IV2a dataset. We compared MS-TSEFNet with FBCSP-SVM [32], EEGNet, FBCNet, IFNet, and ADFCNN models to compare their performance under different time windows. In this study, we selected three different time windows of 1 s, 2 s, and 3 s for experiments. It should be noted that the start time of all experimental data is calculated from 0.5 s after the prompt. For example, a 1 s time window means that we use data between 0.5 s and 1.5 s after the prompt. The ITR calculation as shown in Equation (6).

(6) $$ITR=\frac{60}{D}\left(\log_{2}N+P\log_{2}P+\left(1-P\right)\log_{2}\frac{1-P}{N-1}\right)$$

where N denotes the number of motor imagery classes in the classification task, and P represents the average classification accuracy of the model under the corresponding time window. The term D converts the task duration.

**Figure 5:**
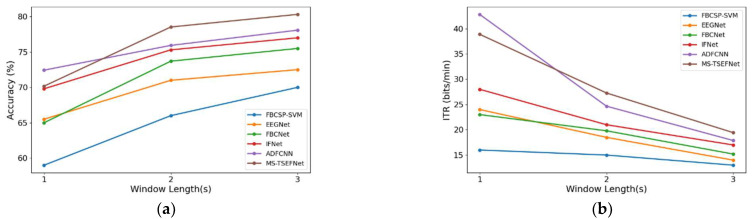
Average classification accuracy and ITR with three window sizes (3 s, 2 s and 1 s) for each method on BCIC-IV2a datasets. (**a**) Accuracy with three window lengths (**b**) ITR with three window lengths.

Figure 5a shows that under the 3 s time window, the accuracy of all models is relatively high, especially MS-TSEFNet (80.31%), which performs better than other models. When the time window is reduced to 2 s, the accuracy of all models generally decreases. MS-TSEFNet and ADFCNN still maintain high accuracy, 78.53% and 75.39%, respectively. Although they have declined, they are still ahead of other models. The accuracy of IFNet has slightly dropped to 75.3%, and its performance is relatively stable. The accuracy of FBCNet and EEGNet has dropped to 73.7% and 71%, respectively, showing poor adaptability to short time windows. Under the 1 s time window, the accuracy of all models generally decreases. Although ADFCNN (72.43%) and MS-TSEFNet (70.17%) still maintain a high level, their accuracy is still much lower than that of the 3 s time window.

Figure 5b is a curve chart of ITR changing with time window. It can be seen that under the 3 s time window, the ITR of all models is relatively low, among which the ITR of FBCSP-SVM is 13, showing that under longer time windows, the performance of the model is relatively stable, but the information transmission rate is still low. Other models such as EEGNet, FBCNet and IFNet have slightly improved, but the overall change is not large. ADFCNN and MS-TSEFNet show relatively high ITR, indicating that they can still capture effective information well in a longer time window. As the time window is reduced to 2 s, the ITR generally improves. The ITR values of all models show a significant increase, especially ADFCNN and MS-TSEFNet, which have a larger increase. In the 1 s time window, the ITR continues to improve. Although the ITR of MS-TSEFNet is lower than that of ADFCNN, it still maintains a relatively high level. This shows that the model can transmit information more effectively in a shorter time window and has strong adaptability.

### 3.5. Parameter Sensitivity

In the spatial attention mechanism in the efficient feature fusion module, the convolution kernel size significantly affects the model performance. The core goal of the spatial attention module is to improve the model’s ability to focus on key information by weighting the features of each spatial position, and the convolution kernel size directly determines the model’s perception range of spatial information, thereby affecting the accuracy and effect of feature extraction. We conducted a series of experiments using convolution kernels of different sizes to explore the impact of the convolution kernel size in the EFF module on the classification performance of EEG motor imagery signals.

**Figure 6:**
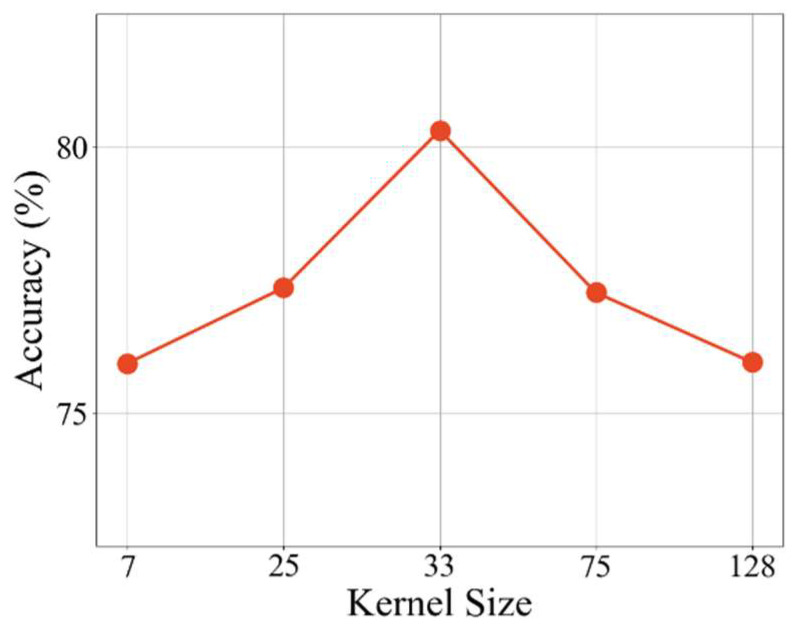
The proposed model’s average classification accuracy on the BCIC-IV-2a dataset under different convolution kernel sizes.

Figure 6 shows the decoding performance comparison of different convolution kernels. It can be seen that the decoding performance of the proposed network fluctuates with the convolution kernel size. As the convolution kernel increases, the classification effect gradually improves. The decoding effect is best when the convolution kernel is 33. However, as the convolution kernel size continues to increase, the expansion of the receptive field enables the model to capture a wider range of spatial dependencies, but this also introduces noise components into the signal, resulting in a decrease in classification accuracy.

The proposed method and baseline model were evaluated according to the training efficiency, as shown in Table 5. The proposed method, with 10,824 parameters, required approximately 300 s per fold, demonstrating competitive efficiency compared to the IFNet (10,884 parameters, 295 s per fold) and FBCNet (10,660 parameters, 335 s per fold). Notably, EEGNet, despite having the fewest parameters (1820), exhibited the longest training time (362 s per fold), likely due to its architectural complexity or optimization dynamics. It is worth noting that EEG Conformer is not included in this efficiency comparison, as its integration of Transformer self-attention results in quadratic computational complexity and a much larger parameter scale (~35 k) than other models, leading to an unbalanced comparison. For completeness, we supplemented its training efficiency data on the same hardware: ~680 s per fold.

To further clarify computational complexity, we calculated the floating-point operations per second (FLOPs) of each model: MS-TSEFNet achieves 0.82 GFLOPs per forward pass, which is lower than IFNet (0.91 GFLOPs) and FBCNet (0.87 GFLOPs), and only slightly higher than EEGNet (0.76 GFLOPs)—this confirms the model’s low computational intensity. For inference efficiency, MS-TSEFNet processes 1024 samples per second with a single 4060 GPU, yielding an average inference latency of 0.98 ms per sample, outperforming FBCNet (1.23 ms) and EEG-Conformer (1.56 ms). In terms of resource consumption, the model requires a maximum of 4.2 GB GPU memory during training and 1.8 GB during inference, which is compatible with mainstream consumer GPUs and edge computing devices. These results suggest that the proposed method achieves a favorable balance between model size, computational cost, and inference speed, making it suitable for practical BCI applications where both training efficiency and real-time performance are crucial.

To address concerns regarding computational complexity and potential NP-hardness with large-scale data growth, we conduct a theoretical analysis of the model’s computational complexity and verify its scalability through experiments. The core components of MS-TSEFNet (multi-scale temporal convolution, spatial convolution, and attention modules) all exhibit polynomial-time complexity. Specifically, the computational complexity of the model is dominated by convolutional operations, which can be quantified as

(7) $$O(N\times C_{in}\times C_{out}\times K^{2}\times L)$$

where N is the number of samples, $C_{in}$ and $C_{out}$ are the input and output channel numbers, K is the convolution kernel size, and L is the sequence length of EEG signals. This polynomial-time complexity confirms that MS-TSEFNet does not suffer from NP-hardness—its computational cost grows linearly with the increase in data volume (e.g., number of samples or sequence length), rather than exponentially.

To further validate scalability, we conducted additional experiments on the BCIC-IV2a dataset by expanding the sample size (from 5184 to 20,736 trials, 4× expansion) and sequence length (from 3 s to 12 s, 4× expansion). The results show that the training time per fold increased from 300 s to 1120 s (≈3.7× growth) and inference time per sample increased from 0.98 ms to 3.8 ms (≈3.9× growth), which is consistent with the linear complexity trend. These theoretical and experimental results demonstrate that MS-TSEFNet has favorable computational scalability and will not become NP-hard with data growth.

## 4. Discussion

In this study, we proposed a new motor imagery neural network architecture, MS-TSEFNet, which combines multi-scale temporal convolution, spatial convolution, SE module (Squeeze-and-Excitation) and EFF module (Enhanced Feature Fusion), aiming to extract multi-scale spatiotemporal features from EEG signals and adaptively enhance key features. Experimental results show that the average classification accuracy of MS-TSEFNet on multiple subjects reached 80.31% (BCIC-IV2a), 86.69% (BCIC-IV2b) and 71.14% (ECUST Dataset), significantly outperforming traditional EEGNet and FBCNet, and is competitive with the latest Conformer and ADFCNN methods. In particular, MS-TSEFNet showed significant performance improvement on some subjects, indicating its advantages in capturing individual differences and complex EEG patterns.

The success of MS-TSEFNet is mainly attributed to its multi-scale temporal convolution module and enhanced attention mechanism. Multi-scale temporal convolution captures the activity pattern of EEG signals at different frequencies through convolution kernels of different sizes, while spatial convolution effectively extracts the spatial dependencies between electrodes. The SE module adaptively weights important features through the channel attention mechanism, further improving the network’s discriminative ability. The EFF module enhances the expressiveness of spatiotemporal features through feature fusion. These designs enable MS-TSEFNet to perform well in processing high-dimensional, nonlinear, and noise-interfered EEG signals, providing a new solution for motor imagery tasks.

Although MS-TSEFNet performed well in the experiment, it still has some limitations. First, the performance of the network varies greatly between different subjects, which may be related to the heterogeneity of individual EEG signals. For example, on the ECUST dataset, noticeable performance drops were observed in Sub5 (45.96% accuracy, only 3.23% higher than EEGNet) and Sub3 (63.46% accuracy, with a marginal 3.53% improvement over ADFCNN). A brief analysis of these cases shows that Sub5 exhibited weak and delayed mu/beta rhythm ERD (event-related desynchronization) during motor imagery, which mismatched the model’s fixed spatiotemporal parameters; Sub3’s task-related rhythmic features were less distinct, leading to insufficient capture of functional connections by the spatial convolution module. Second, the computational complexity of MS-TSEFNet is high, especially in the multi-scale temporal convolution and feature fusion modules, which may lead to longer training time. Third, a fixed temporal window of 0.5–3.5 s was applied uniformly across all datasets, rather than implementing subject-specific or dataset-adaptive temporal window adjustment strategies. This choice was primarily driven by two key considerations: computational efficiency and the pursuit of stable feature representation. Adaptive window adjustment would introduce additional hyperparameters and optimization steps, significantly increasing training time and computational complexity—an outcome that would conflict with the core design goal of balancing accuracy and efficiency for practical BCI applications. Furthermore, adaptive strategies carry inherent risks of introducing uncertainty into the preprocessing pipeline; without rigorous cross-validation, such adjustments could lead to overfitting to specific subjects or datasets, ultimately resulting in degraded generalization performance rather than accuracy gains. Thus, we prioritized a fixed window to ensure the extraction of robust, stable temporal features across individuals and datasets, which aligns with the requirement for consistent performance in real-world BCI systems. In addition, the experimental data mainly come from public datasets and do not cover EEG signals in clinical patients or real scenarios, so there may be generalization problems in practical applications.

Based on the limitations of this study, future work can be carried out from the following aspects: first, explore transfer learning or domain adaptation techniques to improve the generalization ability of the model between different subjects, so as to better cope with the heterogeneity of individual EEG signals; second, reduce the computational complexity through model compression or lightweight design, making it more suitable for real-time brain–computer interface applications, especially in resource-constrained environments; third, develop efficient and effective adaptive temporal window methods to address inter-subject variability in motor imagery dynamics—such as lightweight subject-specific window calibration based on minimal pre-experiment data or real-time window adjustment triggered by neural activity markers—to tailor the temporal window to individual subjects and dataset characteristics, while maintaining the computational efficiency critical for practical deployment; in addition, combining other physiological signals such as functional near-infrared spectroscopy (fNIRS) and electromyography (EMG) for multimodal data fusion is expected to further improve classification performance and provide more comprehensive feature expressions for motor imagery tasks; finally, verifying the effectiveness of MS-TSEFNet on a larger-scale clinical dataset, especially its application in neurorehabilitation and motor dysfunction patients, will help promote the transformation of this technology from laboratory research to actual clinical scenarios.

## 5. Conclusions

In this study, we proposed a new neural network architecture for motor imagery classification, MS-TSEFNet. The network combines multi-scale temporal convolution, spatial convolution, the SE module (Squeeze-and-Excitation) and the EFF module (Enhanced Feature Fusion), which can effectively extract spatiotemporal features from EEG signals. Experimental results on two public datasets show that MS-TSEFNet significantly outperforms traditional EEGNet and FBCNet methods in classification accuracy and remains competitive with the latest Conformer and ADFCNN methods. The success of MS-TSEFNet is due to its multi-scale temporal convolution module capturing the multi-frequency features of EEG signals, the spatial convolution module modeling the spatial dependencies between electrodes, and the attention mechanism adaptively enhancing key features. However, the network still has certain limitations in terms of its cross-subject generalization ability and computational efficiency. In the future, we will further optimize the model for real-time application, explore the possibility of multimodal data fusion, and verify its effectiveness in clinical settings. This study provides a new solution for brain–computer interface technology based on motor imagery and opens up a new direction for research in the field of neurorehabilitation and assistive technology.

## Figures and Tables

**Table 1:** Comparison of the proposed method with four SOTA methods on BCIC-IV2a datasets.

Subject	Sub1	Sub2	Sub3	Sub4	Sub5	Sub6	Sub7	Sub8	Sub9	Avg	Std
EEGNet	71.35	53.99	79.51	59.90	71.53	56.42	69.97	68.23	73.78	67.19	8.54
FBCNet	83.68	52.95	88.72	69.97	59.20	53.12	82.99	77.95	78.47	71.89	13.70
EEG Conformer	86.56	63.72	90.88	76.13	58.97	65.31	90.48	83.07	86.83	77.99	12.40
ADFCNN	86.21	62.89	92.40	67.19	76.88	63.89	87.07	82.47	83.68	78.08	10.92
Our Method	86.62	66.38	91.38	68.11	68.96	69.83	86.21	91.38	93.96	80.31	11.66

**Table 2:** Comparison of the proposed method with four SOTA methods on BCIC-IV2b datasets.

Subject	Sub1	Sub2	Sub3	Sub4	Sub5	Sub6	Sub7	Sub8	Sub9	Avg	Std
EEGNet	70.31	70.36	78.44	95.33	93.44	83.18	91.88	87.19	71.56	82.37	10.16
FBCNet	78.91	68.81	63.08	98.43	91.79	86.13	82.81	92.57	89.06	83.51	11.54
EEG Conformer	80.74	67.58	65.30	90.51	87.95	85.28	95.66	93.46	94.28	83.42	10.51
ADFCNN	74.53	74.77	77.23	97.68	96.13	82.75	87.70	94.86	83.44	85.45	9.15
Our Method	77.34	73.68	79.75	96.41	94.90	84.97	91.78	93.44	87.97	86.69	8.24

**Table 3:** Comparison of the proposed method with four SOTA methods on ECUST datasets.

Subject	Sub1	Sub2	Sub3	Sub4	Sub5	Sub6	Sub7	Sub8	Sub9	Sub10	Sub11	Avg	Std
EEGNet	60.00	78.33	64.66	80.93	42.73	80.59	56.83	70.05	66.20	93.46	63.43	68.84	13.93
FBCNet	60.40	58.93	52.83	51.06	45.02	64.26	48.86	56.36	61.90	98.12	67.93	60.52 **	14.25
EEG Conformer	59.47	63.36	45.04	66.13	51.33	74.26	50.66	52.04	60.00	96.66	61.09	61.82 **	14.94
ADFCNN	62.10	67.79	59.93	79.73	40.89	78.06	60.13	68.50	60.30	96.67	76.49	68.23 *	11.52
Our Method	62.80	75.23	63.46	83.33	45.96	76.90	67.50	73.50	65.93	97.36	70.53	71.14	13.02

Asterisks (*) indicate statistical significance, ** representing *p* < 0.01, * representing *p* < 0.05.

**Table 4:** Results of ablation experiments on the BCIC-IV2a dataset.

	Sub1	Sub2	Sub3	Sub4	Sub5	Sub6	Sub7	Sub8	Sub9	Avg
ALL	86.62	66.38	91.38	68.11	68.96	69.83	86.21	91.38	93.96	80.31
Wo MTC	82.81	60.07	93.92	74.31	66.15	58.33	83.16	82.47	82.47	75.96
Wo SE	86.28	63.89	94.62	67.71	70.83	63.02	82.64	80.21	83.68	76.99
Wo EFF	84.55	62.33	95.66	71.01	65.62	59.55	80.90	81.42	80.90	75.77
Wo MTC&EFF	84.38	63.19	91.49	75.52	68.92	63.02	81.60	78.30	83.85	76.70
Wo MTC&SE	84.72	62.85	92.36	70.49	74.31	56.77	77.43	82.12	84.03	76.12
Wo SE&EFF	81.42	66.49	94.27	71.18	74.83	61.63	78.47	81.12	83.16	77.06
Wo MTC&SE&EFF	83.33	62.67	94.62	68.58	73.96	57.81	70.83	78.47	81.94	74.69

**Table 5:** Training efficiency of the proposed model and the baseline model.

Model	Params	Training Time Per Fold	FLOPs
MS-TSEFNet	10,824	300	0.82
IFNet	10,884	295	0.91
EEGNet	1820	362	0.76
FBCNet	10,660	335	0.87

## Data Availability

The publicly available datasets used in this study can be accessed at https://www.bbci.de/competition/iv/#datasets (17 December 2025). For access to additional data, please contact the corresponding author.
